# Cross paradigm fusion of federated and continual learning on multilayer perceptron mixer architecture for incremental thoracic infection diagnosis

**DOI:** 10.1038/s41598-025-06077-8

**Published:** 2025-07-08

**Authors:** Tianshuo Zhou, Boyuan Wang

**Affiliations:** 1 School of Information Science and Engineering, Lanzhou University, Lanzhou, China; 2 Center for Medical Artificial Intelligence Technology Innovation, Zhuhai Fudan Innovation Research Institute, Zhuhai, China

**Keywords:** Medical imaging, Federated learning, Continual learning, Transnational infectious disease prediction, MLP-mixer, Learning without forgetting, Computer science, Epidemiology, Viral infection

## Abstract

Medical imaging is essential in the study of chest virus infections. Due to data sovereignty issues in healthcare, it is essential to employ federated learning to overcome these obstacles. However, obtaining all relevant data at once is challenging, as it is often acquired incrementally. Therefore, addressing continual learning is imperative. To this end, we combined federated learning with continual learning to construct a transnational infectious disease prediction model. This model was applied to the COVID-XRAY and COVID-CT datasets using 3 and 6 clients, respectively, implementing 4 continual learning algorithms across ten different models. Notably, we integrated MLP-Mixer with Learning without Forgetting (LwF) techniques, achieving an accuracy of 54.34%. This demonstrates the effectiveness of our approach in the early detection, sensing, and timely warning of infectious diseases and ultimately builds a multicentre prediction system for future infectious diseases.

## Introduction

In the current healthcare environment, medical imaging is essential in the intelligent diagnosis of coronavirus infections and other chest virus infections^1^. Rapid and accurate diagnosis of coronavirus and related viral infections is critical to managing and controlling the spread of disease during global epidemics^2^. Medical imaging, particularly through technologies such as X-rays and CT scans, not only plays a key role in the diagnosis of COVID but also in the identification of other chest viral infections. These imaging techniques provide a clear and detailed view of the lungs, facilitating early detection and assessment of the extent of various infections^3^. Intelligent diagnostic systems powered by machine learning and artificial intelligence can analyze these images with speed and accuracy beyond human capabilities^4^. By automating the detection process, these systems significantly reduce the time from symptom onset to diagnosis and treatment, improve patient outcomes, and reduce the burden on healthcare professionals when dealing with various viral respiratory infections^5^. We know infectious diseases have no borders, and our research aims to develop a multinational infectious disease prediction model^6^. The model aims to facilitate early detection, increase early awareness, and provide timely warning of potential outbreaks.

However, due to healthcare data’s high privacy and sensitivity, international data non-sharing poses significant challenges due to data sovereignty issues. To overcome these obstacles, innovative approaches are essential. One such approach is the use of federated learning, which allows the sharing of global models without transferring local data^7^. Through federated learning, organizations can contribute and benefit from collective intelligence to enhance predictive models and medical research while protecting patient privacy. This approach is a significant step forward in overcoming barriers to data sharing, thus advancing global healthcare^8^.

Bian et al. proposed a federated learning method based on pre-trained models (PTMs) that achieved 85% accuracy on the positive prediction and critical illness prediction tasks, and the method performed well in terms of privacy preservation and robustness^9^. Makkar et al. proposed SecureFed, which ensures fairness and robustness, and applied it to the COVID-19 dataset with a prediction accuracy of 88.9%^10^. Sun et al. developed FKD-Med, which fuses FL and KD to achieve up to 1.46% accuracy improvement on the dataset CVC-CLINICDB^11^. Liu et al. proposed FedCL, which integrates contrast learning into federated learning and achieved 93.43% accuracy in COVID-19 chest X-ray dataset^12^. Chanda et al. proposed FedCNNAvg, a federated learning approach using convolutional neural networks (FedCNN) and federated flat (FedAVG), which achieved 98.92% accuracy on the malaria dataset^13^. Qiu et al. developed a joint pseudo-labeling strategy for unlabeled clients via FSSL, with 91.95% of the highest Dice score in the Prostate MRI Segmentation dataset^14^. Wicaksana et al. proposed FedMix, which breaks through the constraints of a single level of image supervision and can dynamically adjust the aggregation weights for each local client, achieving a DC of 91.2 on the Skin tumor segmentation dataset^15^. Hossen et al. proposed FedRSMax, which combines a federated averaging algorithm and randomized client sampling to achieve more than 75% accuracy on the HAM10000 dermoscopy image dataset^16^. Kulkarni et al. developed FedFBN, which incorporates transfer learning and achieves an AUROC of 0.82 on the NIH Chest X-Ray 14 dataset^17^.

Although federated learning has certain applications, existing research rarely considers the issue of incremental data. In real-world scenarios, it is difficult to access all the data at once; data is typically acquired incrementally^18^. Therefore, the problem of continual learning must be addressed. Continual learning, also known as lifelong or incremental learning, is an adaptive algorithm that continuously learns from a stream of information that becomes available over time. This method allows the learning system to adapt to new data and tasks as they emerge without requiring predefined task boundaries^19^. However, during continual learning of multiple tasks, learning new knowledge rapidly destroys previously acquired information i.e., catastrophic forgetting phenomenon, which can lead to a drastic degradation of model performance in the old task^20^. Wang et al. proposed the CoroTrans-CL method for the diagnosis and prevention of severe respiratory diseases caused by coronaviruses. They achieved a co-training accuracy of 95.34% on a dataset containing multiple coronavirus strains^21^. Ye et al. proposed MedCoSS to form a multi-stage pre-training process that was applied to the NCH dataset and achieved an average accuracy of 95.76%^22^. Zhu et al. proposed Triple Enhanced Distillation (TED) to improve knowledge diversity, accuracy, and stability, achieving 88.7% accuracy on the Cardiac Dataset^23^. Gao et al. proposed Incremental learning via counterfactual thinking. The model consists of a feature extractor and a classifier, which achieved 76.59% accuracy on the medical ultrasound classification task dataset^24^. Xiao et al. proposed FS3DCIoT to design a dual-flow modal alignment module, which improved the accuracy by 11.2% on a dermatological diagnostic dataset^25^. Zhang et al. proposed FSCIL-EACA, based on self-supervised learning and modulated attention, which achieved 82.35% accuracy on the HyperKvasir dataset^26^. Tang et al. proposed CLELNet and designed a convolutional self-encoder to extract the representational features of an image, achieving 95.96% accuracy on an esophageal endoscopic image dataset^27^. Zhang et al. proposed ACL to flexibly design learnable lightweight task-specific adapters that achieved an average accuracy of 66.44% on the Skin8 dataset^28^. Hassan et al. proposed the KNC and DAN architectures, which integrate knowledge distillation and attention-enhanced DenseNet structures, achieving a 99.1% accuracy on an acute lymphoblastic leukemia (ALL) detection dataset^29^.

However, current research lacks studies on federated learning under continual learning. To address this gap, we focus on incremental federated learning in cross-regional multi-node scenarios^30^. Specifically, federated learning addresses data privacy issues by processing data locally and sharing only model parameters, ensuring privacy across institutions. Continual learning, on the other hand, handles the challenge of incrementally arriving data, allowing models to learn new tasks without accessing previous data, while minimizing catastrophic forgetting. We designed agents with 3 and 6 local training models, and through federated learning, these models were uploaded to a central server. We proposed a method combining MLP-Mixer with LwF to train on X-ray and CT image datasets of COVID. This method attained an accuracy of 54.34%. By combining these two approaches, we effectively tackle both privacy concerns and the management of incremental data from various sources, paving the way for a robust, privacy-preserving multi-center infectious disease prediction framework.

## Materials and methods

### Dataset

All data utilized in this study were obtained from publicly available open-source online datasets, with no involvement of direct human participation or clinical trials. The COVID-X-ray images were categorized into six classes: Normal (470 images for training and 60 for testing), MERS (135 images for training and 49 for testing), SARS (106 images for training and 27 for testing), Omicron and Delta Variants (111 images for training and 50 for testing), Wild-type SARS-CoV-2 (205 images for training and 52 for testing), and Other Viral Pneumonias (240 images for training and 60 for testing).

The COVID-CT images are categorized into four groups: Normal (954 images for training and 285 for testing), Omicron and Delta Variants (538 images for training and 191 for testing), Wild-type SARS-CoV-2 (450 images for training and 120 for testing), and Other Viral Pneumonias (447 images for training and 118 for testing). These datasets cover various types of viral pneumonia, which are representative of COVID-19 imaging research and aim to simulate the heterogeneity found in real-world multi-center data, despite the limited number of samples in certain categories. They effectively reflect the challenges associated with integrating data across multiple clinical sites.

### Method

#### MLP-Mixer

MLP-Mixer, or Multilayer Perceptron Mixer, is a novel network architecture based on a fully connected layer (MLP), which does not use a convolutional layer or an attention mechanism but instead processes the image through the MLP^31^. The core of the MLP-Mixer consists of two types of MLPs: Channel-mixing MLPs are responsible for mixing information from different channels at each location.

**Fig. 1:**
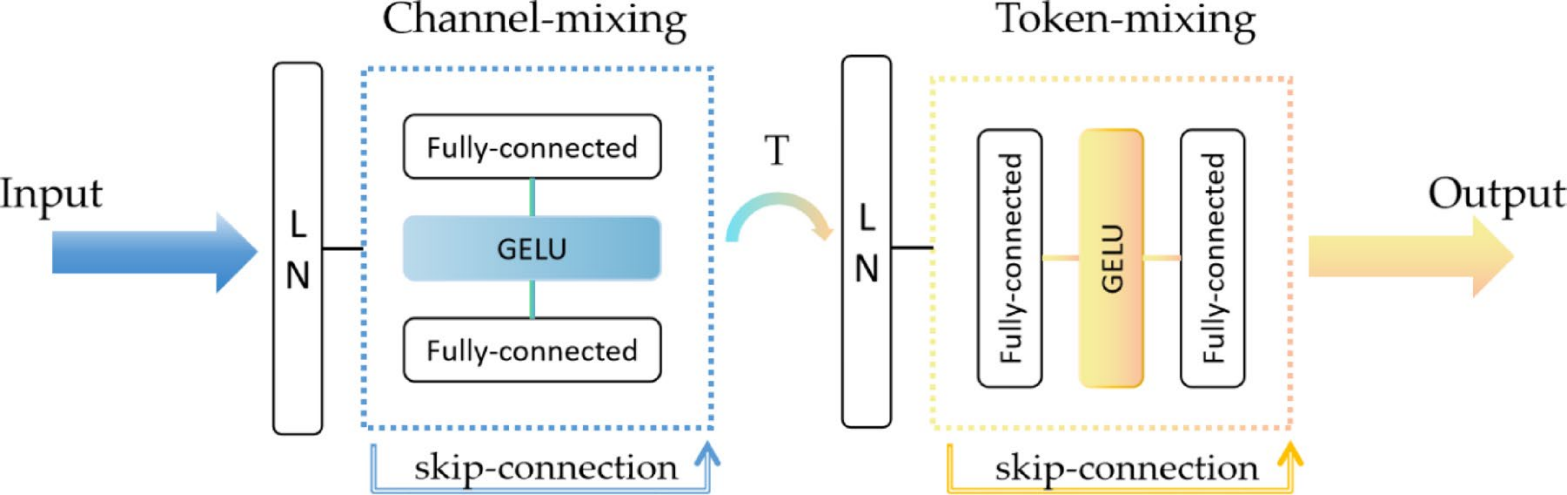
Structure of the MLP-mixer.

Token-mixing MLPs: responsible for mixing information between different locations (tokens), as shown in Fig. 1. For each token, token-mixing is performed first to mix spatial information by fusing neighboring pixels within each token. Then, channel-mixing is performed for each dimension to achieve single-position cross-channel feature fusion. When Mixer combines token-mixing and channel-mixing, it accomplishes the transition from token level to channel level by simply transposing the matrix. The alternation of these two mixing types facilitates the exchange and fusion of information across two dimensions. Additionally, Mixer employs skip-connections to add inputs and outputs and uses Layer Normalization (Layer Norm) before the fully connected layer for pre-normalization. A single MLP consists of two fully connected layers with a GELU activation function in between^32^. The entire computation can be represented as:

1 $$\begin{aligned} \:Z_{{ * \:,i}} & = X_{{ * \:,i}} + W_{2} \times \:\sigma \:(W_{1} \times \:LN(X)_{{ * \:,i}} ),fori = 1...CY_{{ * \:,i}} \\ & = Z_{{ * \:,i}} + W_{2} \times \:\sigma \:(W_{1} \times \:LN\left( {Z)_{{ * \:,i}} } \right),fori = 1...T \\ \end{aligned}$$

In our experiments, we use mixer_b16_224_in21k, where b16 stands for the size of each image block in the model to be 16 × 16 pixels, 224 stands for the size of the input image to be 224 × 224 pixels, and in21k stands for the model to be pre-trained on the ImageNet-21k dataset.

#### Federal learning

Federated learning is a unique machine learning framework enabling multiple participants to train a shared model collaboratively without centralizing their data in one location^33^. This type of learning is crucial for protecting user privacy and data security, as it allows data to remain local, and only necessary model updates are shared to the server for aggregation.

**Fig. 2:**
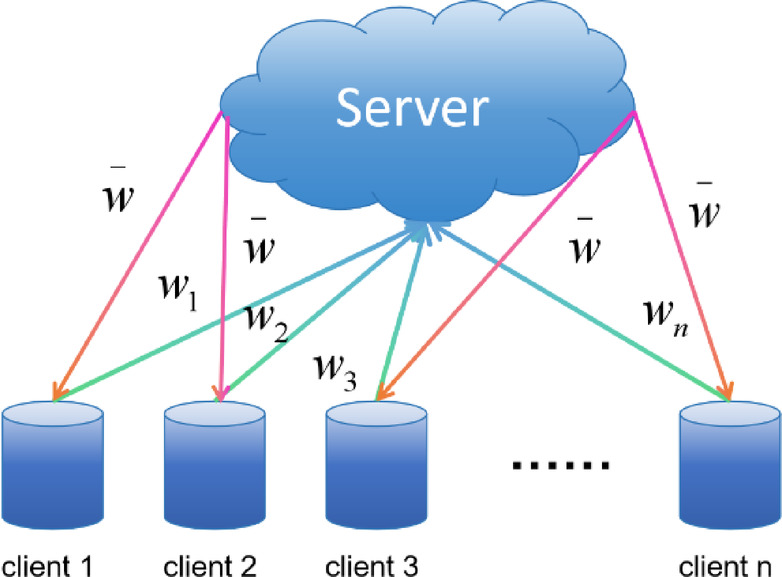
Schematic diagram of the federated averaging (FedAvg) parameter aggregation and distribution process.

FedAvg: FedAvg (Federated Averaging) aggregates model parameters through weighted averaging^34^. The fundamental concept of FedAvg involves uploading the parameters of local models to a server, where the server calculates the average of all the model parameters and then broadcasts this averaged model back to all local devices.as shown in Fig. 2.

The steps follow: the central server initializes a shared model and distributes its parameters to all k participating clients. Each client then independently trains the received model using its data. After training, each client uploads its model parameters (w) back to the central server. After the central server receives updates from all clients, it computes the average of these updates. This step is the key to FedAvg and is calculated as follows:

2 $$\:{w}_{t+1}=\frac{1}{N}{\sum\:}_{k=1}^{K}{n}_{k}{w}_{t+1}^{k}$$

N is the total amount of client data; each client k owns its dataset $\:{D}_{k}$ which is of size $\:{n}_{k}$. The global model is updated using these averaged parameters and then sent back to the clients for the next round of training. These steps are repeated until the model achieves the desired accuracy or other stopping conditions are met.

#### Continual learning

Continual learning starts with an initial non-incremental stage $\:{S}_{0}$ whose model $\:{M}_{0}$ is trained from the dataset $\:{D}_{0}=\{{X}_{i},{Y}_{i};i=\text{1,2},\cdots\:,{P}_{0}\}$. $\:{X}_{i}$ and $\:{Y}_{i}$ denote the set of samples and the set of labels of the ith data class, respectively, and $\:{P}_{0}$ denotes the number of classes trained in the stage $\:{S}_{0}$. For a continual learning process with t stages, it consists of an initial stage and t-1 incremental stages. The incremental phase $\:{S}_{t}$ uses the model $\:{M}_{t-1}$ to train the dataset $\:{D}_{t}=\{{X}_{i},{Y}_{i};i={N}_{t-1}+1,\cdots\:,{N}_{t-1}+{P}_{t}\}$ such that the model can recognize the $\:{N}_{t}={P}_{0}+{P}_{1}+\cdots\:+{P}_{t}$ categories data^35^.

Finetune: Finetune, is usually based on a pre-trained model. This process involves adjusting a model’s parameters by continuing training, typically on a new dataset, to enhance the model’s performance on a specific task. In continual learning, the primary application of this approach is to enable the model to learn new tasks while preserving previously acquired knowledge^36^. Each client simply learns the tasks in sequence.

In the basic finetune process, you have a neural network model pre-trained on some tasks (or multiple tasks), and its parameters are written as θ. Suppose there is a new task, and you wish to continue training this model for this new task while retaining as much as possible of the previously learned knowledge. Given the training data $\:({x}_{i},{y}_{i})$for the new task, we define a loss function $\:L\left(\theta\:\right)$, typically reflecting the performance metric on the new task. The goal of model fine-tuning is to adjust the parameters θ to new values that minimize the loss function:

3 $$\:{\theta\:}^{\ast\:}={arg}{{min}}_{\theta\:}L\left(\theta\:\right)$$

In federated learning, applying fine-tuning allows the model to perform incremental learning for each new task by gradually introducing new classes without forgetting old ones. During each local training round, the model fine-tunes the new tasks using cross-entropy loss and utilizes knowledge distillation (not-tue distillation) to retain knowledge from previous tasks. After each communication round, local updates are aggregated to update the global model. Task performance is continuously tracked to ensure that learning new tasks does not negatively affect the performance on old tasks.

LwF: Learning without Forgetting uses only new task data to train the network while maintaining its original functionality^37^. Relying solely on new task data not only preserves the performance of previous tasks but also acts as a regularization method to enhance the performance of the new task.

We categorize the parameters of the neural network into 3 types: the shared parameter $\:{\theta\:}_{s}$; the parameter $\:{\theta\:}_{o}$on the old task; the specific parameter on the new task and the parameter $\:{\theta\:}_{n}$ that learns to work well in both the new and the old tasks using only the images and labels of the new task. When training, we first freeze $\:{\theta\:}_{s}$ and $\:{\theta\:}_{o}$ and train $\:{\theta\:}_{n}$ until convergence. Then, train all weights $\:{\theta\:}_{s}$,$\:{\theta\:}_{o}$ and $\:{\theta\:}_{n}$ jointly until convergence.

LwF incorporates two objective functions during training: a loss function for the new task and a loss function to maintain the old knowledge. The hybrid loss function can be expressed as:

4 $$\:loss={\lambda\:}_{o}{L}_{old}({Y}_{o}\hat,{{Y}_{o}})+{L}_{new}({Y}_{n}\hat,{{Y}_{n}})+R\hat ({{\theta\:}_{s}}\hat,{{\theta\:}_{o}}\hat,{{\theta\:}_{n}})$$

Losses for new tasks typically use the cross-entropy function, while knowledge distillation losses were used for old tasks.$\:\lambda\:$represents the loss of balance weight between the old and new tasks. Adjusting the performance of the old and new tasks can be modified. R is a regular term.

In federated learning, LwF applies incremental learning for each new task, fine-tuning the model to prevent catastrophic forgetting. After each task, the current model is frozen as the old network to retain knowledge from previous tasks. New tasks are trained locally using cross-entropy loss, while knowledge distillation is applied to integrate the knowledge from the old tasks. After each communication round, the local models are aggregated to update the global model.

EWC: Elastic Weight Consolidation is based on Bayesian online learning, which allows the network to retain knowledge about old tasks while learning new ones through elastic consolidation of weights^38^. When moving from task A to task B, the network aims to minimize the new loss function while imposing specific constraints on the parameters learned from the old task. This is accomplished using the following loss function:

5 $$\:L\left(\theta\:\right)={L}_{B}\left(\theta\:\right)+{\sum\:}_{i}\frac{\lambda\:}{2}{F}_{i}({\theta\:}_{i}-{\theta\:}_{A,i}^{\ast\:}{)}^{2}$$

$\:{L}_{B}\left(\theta\:\right)$ is the loss function for the new task B. $\:{\theta\:}_{i}$ is the current value of the parameter, while $\:{\theta\:}_{A,i}^{\ast\:}$ is the value of the parameter obtained after task A. $\:{F}_{i}$ is the Fisher information about the parameter $\:{\theta\:}_{i}$, which measures the importance of the parameter $\:{\theta\:}_{i}$ in task A. $\:\lambda\:$ is a hyperparameter that controls the influence of the old task on learning the new task. The Fisher information matrix F is the expected value of the variance of the loss function L concerning the gradient of the parameter θ:

6 $$\:{F}_{i}=E\left[(\frac{\partial\:{L}_{A}}{\partial\:{\theta\:}_{i}}{)}^{2}\right]$$

Suppose a parameter is critical in the old task. In that case, the value of the Fisher information for this parameter will be large, and the EWC method will reduce the variation of these parameters by increasing the corresponding regularization term in the loss function.

When applying the EWC to federated learning, the model learns new tasks through incremental training while protecting the knowledge of old tasks via EWC loss. After each task, the model’s parameter mean and Fisher information matrix are saved, and a penalty term is computed to prevent excessive changes to important parameters. Through local training and EWC regularization, the learning of new tasks is balanced with the retention of knowledge from old tasks. Additionally, after each communication round, the locally updated model weights are aggregated, ensuring the global model can effectively adapt to all tasks and avoid catastrophic forgetting.

BEEF: Bi-compatible class-incremental Learning via Energy-Based Expansion and Fusion combines the expansion and optimization of energy functions and the integration of old and new knowledge to solve the problem of “catastrophic forgetting” in incremental learning^39^. BEEF introduces the energy function $\:E(x;{\theta\:}_{y})$, which measures the degree of match between a sample and its corresponding category. When the model encounters a new class, by minimizing the energy value of the sample under the correct class, the model can dynamically expand and adapt to the new class. At the same time, to prevent forgetting the old class when introducing a new class and keep the energy levels of the old and new classes balanced, BEEF designs an objective function:

7 $$\:{{min}}_{\theta\:}{\sum\:}_{i=1}^{N}{E}_{({x}_{i},{y}_{i})}\left[E\right({x}_{i};{\theta\:}_{{y}_{i}}\left)\right]+\lambda\:{\sum\:}_{j=1}^{K}{E}_{{x}_{j}\sim{p}_{j}}[E({x}_{j};{\theta\:}_{{y}_{j}})]$$

In addition, BEEF ensures that introducing new classes does not negatively affect the old classes through a dual compatibility mechanism, maintaining the overall stability of the model. To better integrate the old and new knowledge, BEEF employs a fusion mechanism that combines the energy functions of the old and new classes, thus realizing a smooth transition between the old and new knowledge.

8 $$\:\stackrel{\sim}{E}(x;\theta\:)=\alpha\:{E}_{old}(x;{\theta\:}_{old})+(1-\alpha\:){E}_{new}(x;{\theta\:}_{new})$$

In federated learning, BEEF applies incremental learning by fine-tuning the model for each new task while preserving knowledge from previous tasks. During local training, the model uses cross-entropy loss for new tasks and an energy-based regularization loss to prevent excessive parameter updates that could disrupt previously learned tasks. The energy is calculated by analyzing the magnitude of parameter updates, and a fusion approach is used to blend the old and new energy values, ensuring a balance between new learning and knowledge retention. After each communication round, local updates from clients are aggregated to update the global model.

#### Evaluation indicators

Task accuracy is very important in continual learning tasks based on federated learning. We use the same average accuracy metric as previous studies to evaluate the model’s performance^40^. We define it as the model’s accuracy on the kth task test set after learning i tasks sequentially.

The average accuracy $\:{A}_{i}$ measures the performance of the continual learning algorithm across all test sets after learning i tasks and can be expressed as follows:

9 $$\:{A}_{i}=\frac{1}{i}{\sum\:}_{k=1}^{i}{a}_{i,k}$$

When the amount of test data for each class differs, a weighted version of Eq. (9) is required.

Additionally, to study the issue of catastrophic forgetting in continual learning, we introduce the forgetting measure F^41^. F is an important metric for quantifying the extent to which a model forgets old task knowledge after learning new tasks. To comprehensively evaluate the model’s ability to retain knowledge, we define the forgetting measure F as the average forgetting across all previous tasks after learning the k-th task.

The forgetting measure F_k_ is computed using the following formula:

10 $$\:{F}_{k}=\frac{1}{k-1}{\sum\:}_{j=1}^{k-1}{f}_{k}^{j}$$

where $\:{F}_{k}$ represents the average forgetting of the previous $\:k-1$ tasks after learning the k-th task, and $\:{f}_{k}^{j}$ quantifies the forgetting of the j-th task after learning the k-th task.

Next, to compute the forgetting for each task, we use the following formula:

11 $$\:{f}_{k}^{j}=\frac{1}{\left|{C}_{j}\right|}\sum\:_{c\in\:{C}_{j}}\underset{t\in\:\left\{1,\cdots\:,N-1\right\}}{\text{max}}\left({A}_{c}^{\left(n\right)}-{A}_{c}^{\left(N\right)}\right)$$

where $\:{C}_{j}$ is the set of classes related to the j-th task, $\:{A}_{c}^{\left(n\right)}$ is the accuracy on class c during the n-th learning phase, and $\:{A}_{c}^{\left(N\right)}$ is the final accuracy on class c after all tasks have been learned. By comparing the accuracy differences across learning phases, we can quantify the forgetting of the j-th task.

### Experiment

**Fig. 3:**
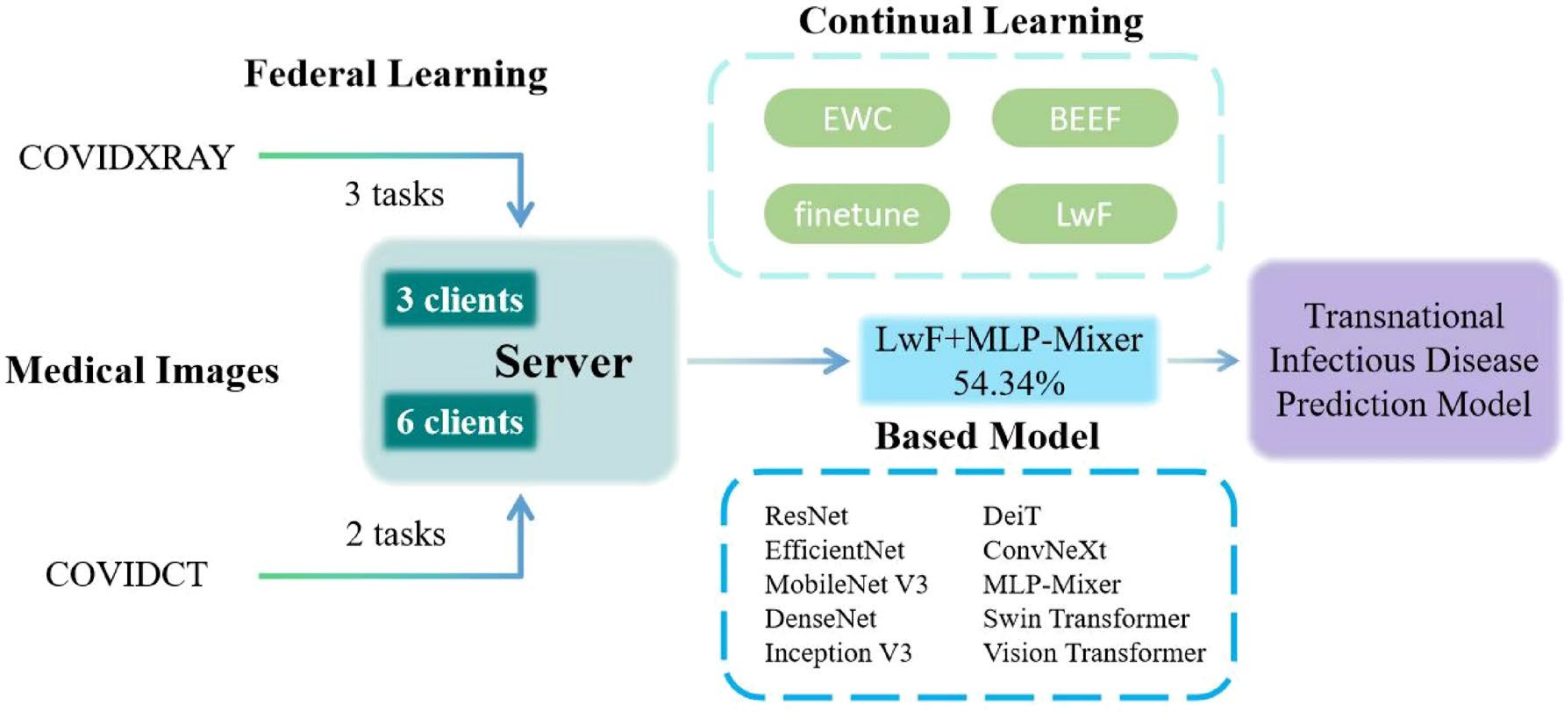
Framework and procedure of federated continual learning for transnational infectious disease prediction.

In this experiment, we established a novel Federated Continual Learning framework for infectious disease prediction and simulated a Transnational Infectious Disease Prediction Model based on this framework as shown in Fig. 3. This model realizes a multi-client-participatory federated learning system with a focus on data privacy. The system simulates 3 and 6 internatioanal clients, all connected to a central server. These clients may represent different healthcare institutions or data centers, which collaboratively participate in building an infectious disease prediction model through federated learning.

On the server side, various machine learning strategies are employed to optimize the performance of the model. Continual learning strategies such as EWC, LwF, BEEF and Finetune are integrated to enhance the model’s adaptability and efficiency. By incorporating these techniques, the model is capable of processing data from diverse clients while ensuring data privacy and guaranteeing the accuracy^42^. Multiple models have been utilized within the system for performance evaluation. They are 10 pre-trained models transferred and fine-tuned from the timm library, allowing for comprehensive analysis and optimization of the predictive capabilities:

Swin Transformer: Swin Transformer is a Transformer-based visual model that uses a localized attention mechanism called “windowing,” making it more efficient in processing large images^43^. In our experiments, we use the model swin_small_patch4_window7_224.

ResNet: ResNet is a Convolutional Neural Network (CNN) architecture that is part of the Residual Network (ResNet) family. It achieves this by introducing so-called “residual chunks”, which effectively allow the network to learn a constant mapping, which helps to transfer information between layers and avoids the problem of vanishing gradients problem^44^. In our experiments, we use the model resnet18.

EfficientNet: EfficientNet is an efficient convolutional neural network architecture designed based on an idea known as the “composite scaling method”, which systematically investigates the scaling of the network’s width, depth, and resolution to find an equilibrium that allows the model to achieve optimal performance under different computational budgets^45^. In our experiments, we use the model efficientnet_b0.

MobileNet V3: MobileNetV3 is an efficient convolutional neural network architecture optimized for mobile and edge devices that automatically finds the optimal structure for a given hardware through Network Architecture Search (NAS)^46^. In our experiments, we use the model mobilenetv3_large_100.

Vision Transformer: Vision Transformer (ViT) is an innovative image processing model that applies the self-attention mechanism (with Transformer as the core component) to the field of computer vision^47^. In our experiments, we use the model vit_base_patch16_224.

ConvNeXt: ConvNeXt is based on design insights from the Transformer architecture and improves the performance and efficiency of the model by redesigning and optimizing key components in standard convolutional networks^48^. In our experiments, we use the model convnext_base.

DenseNet: DenseNet is a convolutional neural network architecture characterized by dense connections between each layer. This design significantly enhances gradient propagation, reduces the number of parameters, and increases the network’s efficiency and effectiveness^49^. In our experiments, we use the model densenet121.

Inception V3: Inception V3 is an advanced convolutional neural network architecture that can capture different scale features of an image within the same layer by introducing multi-scale processing techniques and convolutional decomposition to improve feature representation^50^. In our experiments, we use the model inception_v3.

DeiT: DeiT (Data-efficient Image Transformers) is a Transformer model designed for image categorization that improves data efficiency using knowledge distillation techniques, allowing the model to learn effective visual representations even with less data^51^. In our experiments, we use the model deit_small_patch16_224.

There is also the MLP-Mixer model, which we described earlier. In our experiments, we use the model mixer_b16_224_in21k.

Our experiments were conducted on two 4090 GPUs, where we set 3 incremental learning tasks for COVIDXRAY(6 classes) and 2 for COVIDCT(4 classes). We configured the experiment for federated learning with both 3 and 6 clients. Each client was set to undergo 32 local training epochs, with 15 communication rounds. As for the dataset, we performed data transformations on the pre-split training and test sets, including resizing and normalization, and conducted label mapping.

In our experiment, data partitioning was done using a Non-IID (Non-Independent and Identically Distributed) approach, specifically using the Dirichlet distribution to allocate the data^52^. By setting beta to 0.5, the data is not evenly distributed across clients; instead, there are variations in the data distribution between clients. Each client’s dataset contains samples from multiple classes, but the number of samples per class varies. The task partitioning and incremental learning process is carried out by progressively introducing new classes. We first determine the number of classes for the initial task, and these classes are trained in the model first. Subsequently, each task introduces more classes, allowing the model to gradually encounter more classes and adapt to them. The training for each task is done independently, and as the number of classes increases, the dataset for each task expands, ensuring that each task builds on the previous one with new learning objectives.

In the process of local training and global aggregation, each client independently trains its model and updates its weights. These local updates are periodically sent to a central server. Upon receiving the local models from the clients, the server aggregates the weights. The server averages the model weights from all clients, resulting in an updated global model, which is then sent back to the clients. The clients receive the global model and continue with the next round of local training. This process is repeated, with each round involving local training and global aggregation to gradually improve the model’s performance.

## Results

In this study, we used 4 key metrics to evaluate the performance of different models and methods in incremental learning, with the results shown in Tables 1, 2, 3 and 4. Step1 indicates the accuracy at the end of the first task. Step2 indicates the model’s overall accuracy after the second task is added. F (Forgetting measure) measures the extent to which the model forgets previously learned tasks as new tasks are introduced. It quantifies the accuracy of old tasks after each new task is added, reflecting the model’s ability to retain old knowledge during the incremental learning process. Avg denotes the average accuracy of all tasks, a comprehensive indicator of overall performance. Combining the analyses of these metrics, we draw the following conclusions.

**Table 1:** Average accuracy and Forgetting of 3 clients using different CL algorithms and CNN-based models (%).

Models	Parameters	CL methods	X-ray				CT		
			Step1	Step2	Avg	F	Step1	Avg	F
ResNet	11.7 M	Finetune	85.32	33.18	38.59	45.34	99.68	39.92	99.68
		EWC	92.66	24.64	38.59	53.91	99.04	26.75	37.63
		BEEF	95.41	38.39	40.27	43.12	98.71	37.68	96.78
		LwF	95.41	39.81	37.92	47.82	99.04	39.92	99.04
MobileNet V3	5.4 M	Finetune	95.41	42.18	24.5	28.44	100.0	33.61	63.99
		EWC	96.33	23.22	16.44	25.69	100.0	43.56	72.03
		BEEF	98.17	45.97	23.15	28.9	100.0	26.89	83.92
		LwF	93.58	42.65	43.96	33.47	96.78	35.29	25.08
EfficientNet	5.3 M	Finetune	94.5	43.13	34.56	34.87	66.24	36.13	32.16
		EWC	97.25	23.22	16.44	26.15	69.77	38.66	31.83
		BEEF	96.33	45.02	29.53	32.03	49.2	24.79	10.94
		LwF	97.25	49.29	37.25	39.73	46.95	31.65	8.36
DenseNet	8.0 M	Finetune	84.4	45.5	32.89	27.52	100.0	40.2	59.81
		EWC	92.66	23.22	16.44	23.86	100.0	26.75	38.59
		BEEF	84.4	35.55	36.91	17.89	100.0	58.54	29.58
		LwF	77.06	37.44	39.93	30.53	100.0	50.14	3.54
Inception V3	27.2 M	Finetune	85.32	30.81	24.16	15.6	98.71	45.52	19.29
		EWC	78.9	34.12	16.44	38.55	99.68	26.75	38.27
		BEEF	91.74	36.49	28.86	17.49	63.67	36.83	2.26
		LwF	91.74	36.49	28.86	23.85	100.0	45.52	0.64
ConvNext	88.6 M	Finetune	89.91	23.7	27.18	67.14	99.36	44.26	79.75
		EWC	88.99	27.96	28.52	63.16	95.5	26.75	34.09
		BEEF	79.82	25.59	31.88	27.13	98.07	41.88	5.14
		LwF	85.32	36.97	31.21	42.74	99.68	45.1	25.4

**Table 2:** Average accuracy and Forgetting of 3 clients using different CL algorithms and Transformer-based and MLP-Mixer models (%).

Models	Parameters	CL methods	X-ray				CT		
			Step1	Step2	Avg	F	Step1	Avg	F
Swin transformer	49.6 M	Finetune	90.83	33.18	36.58	45.19	100.0	44.68	75.56
		EWC	96.33	20.38	32.89	54.98	99.68	46.64	63.67
		BEEF	86.24	35.55	35.91	59.21	100.0	49.86	47.91
		LwF	88.07	35.07	32.55	37.53	100.0	48.6	28.3
Vision transformer	86.4 M	Finetune	90.83	24.64	30.2	65.4	99.68	42.86	79.74
		EWC	87.16	25.12	29.87	65.25	99.68	38.52	19.94
		BEEF	81.65	25.12	28.52	71.79	99.68	48.6	52.09
		LwF	87.16	43.13	41.61	32.56	99.68	40.9	29.91
DeiT	22.1 M	Finetune	92.66	24.64	27.85	65.7	99.68	44.12	79.75
		EWC	88.07	24.64	31.54	63.53	99.68	46.5	35.69
		BEEF	82.57	27.96	33.56	64.85	99.68	49.58	30.87
		LwF	85.32	45.5	40.6	35.92	99.68	36.83	41.8
MLP-mixer	59.9 M	Finetune	95.41	27.01	36.91	61.46	100.0	52.8	94.53
		EWC	96.33	26.54	35.57	61.47	100.0	50.56	81.99
		BEEF	96.33	50.71	40.6	58.98	100.0	49.44	82.64
		LwF	96.33	43.6	**49.66**	**21.85**	100.0	47.48	**8.04**

**Table 3:** Averag﻿e accuracy and Forgetting of 6 clients using different CL algorithms and CNN-based models (%).

Models	Parameters	CL methods	X-ray				CT		
			Step1	Step2	Avg	F	Step1	Avg	F
ResNet	11.7 M	Finetune	48.62	31.28	29.87	18.35	100.0	39.92	100
		EWC	52.29	23.22	16.44	3.67	100.0	26.75	38.59
		BEEF	72.48	24.17	34.9	46.06	100.0	38.1	100
		LwF	82.57	30.33	35.23	44.79	100.0	46.92	81.35
MobileNet V3	5.4 M	Finetune	90.83	42.18	26.85	33.51	48.23	43.42	30.55
		EWC	90.83	23.22	16.44	22.94	98.71	26.75	37.3
		BEEF	90.83	29.38	40.27	20.19	91.64	40.62	68.17
		LwF	90.83	34.6	33.22	49.26	100.0	41.88	40.51
EfficientNet	5.3 M	Finetune	91.74	45.02	44.97	25.53	44.05	44.12	19.29
		EWC	94.5	26.07	36.58	25.16	66.24	26.75	4.83
		BEEF	91.74	34.12	26.85	38.53	45.34	35.85	8.04
		LwF	94.5	36.49	40.6	51.16	65.59	16.81	27
DenseNet	8.0 M	Finetune	76.15	30.81	40.6	20.19	99.36	56.3	44.7
		EWC	85.32	23.22	16.44	20.19	100.0	26.75	38.59
		BEEF	77.98	29.38	39.6	29.36	100.0	58.4	65.27
		LwF	79.82	47.39	44.97	15.92	74.28	46.78	7.07
Inception V3	27.2 M	Finetune	80.73	40.76	27.85	30.89	65.59	59.1	14.46
		EWC	77.06	23.22	16.44	16.06	52.41	26.75	0
		BEEF	85.32	35.55	30.87	23.95	61.41	47.2	0
		LwF	67.89	33.65	37.58	20.5	100.0	51.96	1.93
ConvNext	88.6 M	Finetune	79.82	23.22	29.87	54.97	99.04	33.61	67.53
		EWC	79.82	24.17	29.53	55.06	98.07	39.5	30.55
		BEEF	88.99	25.12	38.26	52.77	94.21	39.78	33.12
		LwF	79.82	42.65	38.26	30.69	98.71	40.9	46.3

**Table 4:** Average accuracy and Forgetting of 6 clients using different CL algorithms and transformer-based and MLP-mixer models (%).

Models	Parameters	CL methods	X-ray				CT		
			Step1	Step2	Avg	F	Step1	Avg	F
Swin transformer	49.6 M	Finetune	86.24	38.86	39.26	39.84	99.68	46.64	82.64
		EWC	86.24	28.91	35.23	44.86	100.0	51.96	70.42
		BEEF	96.33	29.86	33.22	43.26	99.68	47.76	87.46
		LwF	86.24	42.18	41.61	19.59	100.0	54.06	27.01
Vision transformer	86.4 M	Finetune	81.65	35.07	35.57	28.57	99.68	40.48	51.77
		EWC	83.49	29.38	33.22	37.74	99.68	46.5	24.76
		BEEF	95.41	24.64	28.52	61.42	99.68	36.13	20.26
		LwF	86.24	39.81	38.59	21.17	99.68	40.48	29.26
DeiT	22.1 M	Finetune	82.57	31.75	31.88	37.65	99.68	45.94	83.6
		EWC	79.82	28.44	32.21	55.98	99.68	46.08	40.52
		BEEF	96.33	25.12	31.54	56.99	99.68	47.06	22.51
		LwF	81.65	44.08	42.95	27.94	99.68	41.88	32.16
MLP-mixer	59.9 M	Finetune	96.33	55.45	38.59	58.01	100.0	48.18	81.03
		EWC	96.33	53.55	41.95	51.17	100.0	48.6	77.49
		BEEF	96.33	27.49	36.91	52.84	100.0	49.86	83.28
		LwF	96.33	50.71	**53.02**	**22.36**	100.0	**54.34**	23.47

Among the four continual learning algorithms, LwF and BEEF perform significantly better than Finetune and EWC, with EWC even performing worse than Finetune, possibly due to federated learning effects. Specifically, CNN-based models achieve average accuracies of around 20–40%. Meanwhile, transformer-based models perform slightly better, with accuracies ranging from 30 to 50%. The most effective MLP-Mixer can achieve over 50% accuracy when combined with the LwF algorithm. While this accuracy is not very high, it is important to consider the inherent challenges of medical imaging, particularly in viral infection detection. Chest X-rays and CT images often have weak features and high inter-institutional data heterogeneity. Even without the continual learning constraints, our highest accuracy on the same federated learning setup barely exceeds 60%. With the introduction of continual learning, the issue of catastrophic forgetting further limits performance. Additionally, similar studies have reported lower accuracies in federated continual learning setups. It is worth noting that Finetune and BEEF work well on the COVIDCT dataset with the DenseNet model, achieving an average accuracy close to 60%. Still, their performance on the COVIDXRAY dataset is mediocre.

**Fig. 4:**
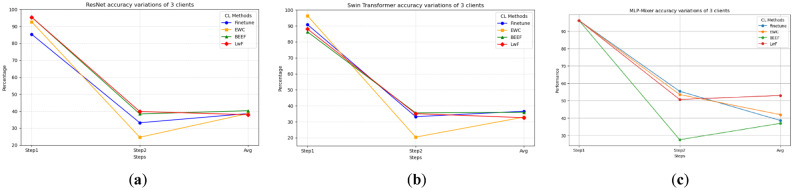
Task accuracy variations of 3 clients: (**a**) CNN models task accuracy variations; (**b**) Transformer-based model task accuracy variations; (**c**) MLP-mixer task accuracy variations.

**Fig. 5:**
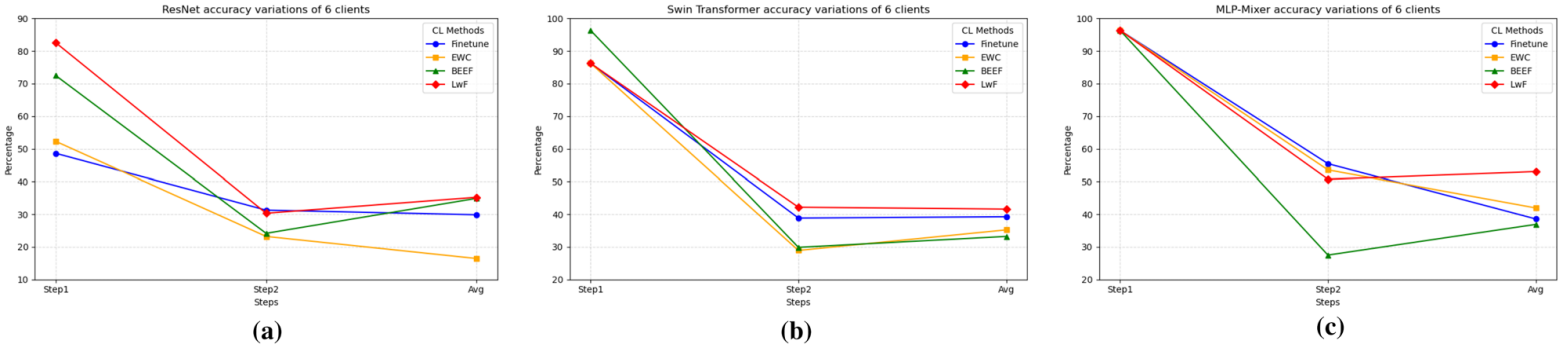
Task accuracy variations of 6 clients: (**a**) CNN models task accuracy variations; (**b**) Transformer-based model task accuracy variations; (**c**) MLP-Mixer task accuracy variations.

From the perspective of accuracy changes at each step of continual learning, as shown in Figs. 4 and 5, LwF performs best in mitigating catastrophic forgetting. In the accuracy change between Step 1 and Step 2, LwF shows less decline, indicating that the model’s overall performance stays better after adding new tasks. The forgetting (F) metric for LwF also indicates effective retention of old knowledge, as it shows lower values, meaning less catastrophic forgetting compared to other methods. The model efficiently retains old task knowledge while learning the new task, avoiding severe forgetting.

The performance of the BEEF method in continual learning is relatively robust. In terms of forgetting (F), BEEF shows good performance with low forgetting, retaining a significant portion of old knowledge, while demonstrating strong adaptability to new tasks. Its overall performance is stable and relatively high.

EWC also performs well in terms of forgetting. Although there is some decline in accuracy between Step 1 and Step 2, the forgetting (F) metric for EWC remains low, indicating that it effectively avoids catastrophic forgetting and retains old task knowledge well, even in the presence of data heterogeneity.

In contrast, the Finetune method performs the worst. While it performs better on new task learning, the forgetting (F) metric shows high values, indicating severe catastrophic forgetting of old knowledge. Finetune adapts quickly to new tasks, but its inability to retain old knowledge leads to significant forgetting and overall poor performance.

From the model perspective, Transformer-based models and MLP-Mixer perform well in processing global information and complex patterns and can better balance old and new task knowledge. In contrast, traditional CNN models perform well in local feature extraction but have relatively weak retention of old knowledge in incremental learning.

The average accuracy of the 6 clients is overall better than that of the 3 clients. As we know that medical data is highly private and there are six World Health Organization’s (WHO) regions, we hope to simulate the uploading of medical data from each region by modelling 6 clients to build a multinational predictive model of infectious diseases for early detection, early sensing, and timely warnings, which will help to improve the health of everyone around the world.

## Discussion

The analysis of task accuracy changes showed that LwF performed the best, followed by BEEF, while Finetune and EWC performed less well. LwF was more effective in balancing the retention of old and new knowledge and did not lose the memories of the previous task while learning the new task. This is because LwF effectively mitigates catastrophic forgetting when dealing with incremental learning by using knowledge distillation to retain the knowledge of the old task during the learning of the new task.

The BEEF approach shows a more robust performance in continual learning. Although it is not as good as LwF in the retention of old knowledge, it maintains high stability through an effective parameter updating strategy. It can avoid catastrophic forgetting to a certain extent while showing good adaptability in new task learning. In contrast, although Finetune performs better in new task learning, it performs poorly in old knowledge retention, which can easily lead to catastrophic forgetting. The main reason is that Finetune trains directly on new tasks, which easily leads to large changes in the model’s parameters, thus losing the knowledge of the previous tasks. EWC, on the other hand, tries to retain the important knowledge of the old tasks in new task learning by adding a regular term for the importance of the parameters. However, in the Federated Learning environment, due to the distributional nature of the data and heterogeneity, the EWC’s efficiency is limited, leading to its poor performance in retaining old knowledge.

The results clearly show the significant difference in performance between the different models. Traditional CNN models are mainly good at extracting local features of an image, which performs well when dealing with routine image classification tasks^53^. However, these CNN models may not perform well when dealing with tasks that require capturing global information and complex patterns of images, especially in incremental learning scenarios of chest virus-infected images, which tend to contain more subtle and critical variations and require models with stronger global understanding^54^. In addition, the performance of these CNN models is also affected to some extent by the size of their number of parameters. For example, models with a smaller number of parameters, such as MobileNet and EfficientNet, while having advantages in terms of lightweight and processing speed, may not be able to capture global information as well as models with a larger number of parameters, such as DenseNet and Inception V3 when faced with complex incremental learning tasks. expressive power, they also entail higher computational costs and the potential risk of overfitting, especially in multi-client federated learning environments.

In contrast, Transformer-based models designed to optimize sequence data processing are particularly adept at capturing long-range dependencies. Its self-attention mechanism can effectively integrate information from the entire image, making such models better handle medical image analysis tasks requiring global information integration^55^. This is particularly important in incremental learning tasks under the federated learning framework, where models are required to learn efficiently on continuously updated data without forgetting established knowledge. In addition, MLP-Mixer, an emerging architecture, supports information processing across spatial locations through its unique multilayer perceptron processing. This design may show advantages in analyzing chest viral infection images. It can better capture and integrate key information from unstructured medical image data, especially when employing the LwF algorithm for incremental learning. MLP-Mixer shows high flexibility and efficiency.

We know that the average correctness of 6 clients is overall better than that of 3 clients. Increasing the number of clients brings higher data diversity, allowing the model to learn from a broader range of data features, thereby improving generalization. Additionally, having more clients helps reduce the risk of model overfitting and enhances the model’s ability to adapt to different data characteristics; in addition, multiple clients provide richer information and enhance the model’s error tolerance and robustness.

## Conclusions

We utilized federated learning to construct a multinational infectious disease prediction model by sharing a global model locally without transferring data across borders. This approach was applied to the COVIDXRAY and COVIDXCT datasets using 3 and 6 clients, respectively. Notably, the use of six clients is particularly significant as it mirrors the WHO six-region distribution, facilitating a simulation that closely represents global medical data processing and enhances the relevance and applicability of our findings to real-world scenarios. This setup effectively addresses the privacy and sensitivity concerns associated with medical data. Considering the challenge of incremental data acquisition, we implemented 4 continual learning algorithms across ten different models to mitigate catastrophic forgetting, thus filling a significant research gap in incremental federated learning across multi-regional, multi-node scenarios. Our innovative integration of MLP-Mixer and LwF techniques achieved a precision rate of 54.34%, demonstrating the effectiveness of our method in early detection, sensing, and timely warnings of infectious diseases. However, our study also has limitations. We acknowledge that current methods face challenges in terms of model stability and knowledge retention under highly heterogeneous data distributions and task environments. For example, when there are significant differences in client data distributions, FedAvg may suffer from reduced generalization ability. Additionally, when faced with large-scale task shifts or class imbalance, continual learning methods may still experience catastrophic forgetting. Nevertheless, through a systematic comparison of multiple representative continual learning methods and experimental analysis under a federated setting, we have laid the foundation for building a more stable and robust multi-center incremental learning system. In the future, more focus should be placed on exploring the applicability of these methods in real-world applications and how to further optimize federated learning and continual learning techniques to achieve more efficient and stable disease prediction models.

## Data Availability

The data presented in this study are available on request from the corresponding author.
